# Tunable superconducting diode effect in a topological nano-SQUID

**DOI:** 10.1126/sciadv.adw4898

**Published:** 2025-09-19

**Authors:** Ella Nikodem, Jakob Schluck, Max Geier, Michał Papaj, Henry F. Legg, Junya Feng, Mahasweta Bagchi, Liang Fu, Yoichi Ando

**Affiliations:** ^1^ Physics Institute II, University of Cologne, Zülpicher Str. 77, 50937 Köln, Germany.; ^2^ Department of Physics, Massachusetts Institute of Technology, Cambridge, MA 02139, USA.; ^3^ Department of Physics and Texas Center for Superconductivity at the University of Houston (TcSUH), Houston, TX 77204, USA.; ^4^ Department of Physics, University of Basel, Klingelbergstrasse 82, 4056 Basel, Switzerland.; ^5^ SUPA, School of Physics and Astronomy, University of St. Andrews, North Haugh, St. Andrews KY16 9SS, UK.

## Abstract

A Josephson diode passes current with zero resistance in one direction but is resistive in the other direction. While such an effect has been observed in several platforms, a large and tunable Josephson diode effect has been rare. Here, we report that a simple device consisting of a topological-insulator (TI) nanowire side-contacted by superconductors to form a lateral Josephson junction presents a large diode effect with the efficiency η reaching 0.3 when a parallel magnetic field $B_{\|}$ is applied. The sign and the magnitude of η are tunable not only by $B_{\|}$ but also by the back-gate voltage. This diode effect can be understood by modeling the system as a nano–superconducting quantum interference device (SQUID), in which the top and bottom surfaces of the TI nanowire each form a line junction and $B_{\|}$ creates a magnetic flux to thread the SQUID loop. This model further shows that the observed diode effect marks the emergence of topological superconductivity in TI nanowire–based Josephson junction.

## Teaser

A large tunable superconducting diode effect is found in a side-contacted topological-insulator nanowire Josephson junction.

## INTRODUCTION

Superconducting (SC) diode effects have become a topic of current interest (*1*, *2*), mainly because they allow for the realization of useful SC circuit components for low-dissipation electronics with potential applications in quantum computing (*3*, *4*) and SC neural networks (*5*). Both bulk superconductors and Josephson junctions may present a diode effect when inversion and time-reversal symmetries are broken, and the latter is called Josephson diode effect (*2*, *6*). A wide range of mechanisms can give rise to an SC diode effect, including asymmetric vortex pinning in a bulk SC slab (*7*–*9*), Meissner screening (*10*), asymmetric current-phase relationship in a junction (*11*–*14*), magnetochiral anisotropy (*1*, *15*, *16*), finite-momentum pairing (*17*–*20*), and topological phase transitions (*21*–*23*).

The Josephson diode effect is of particular interest because of its potential for high controllability (*14*). One way to achieve a highly tunable device with a high diode efficiency is to make an asymmetric superconducting quantum interference device (SQUID) involving two or more Josephson junctions (*12*, *13*). We have recently found (*24*) that a single Josephson junction consisting of a topological-insulator (TI) nanowire side-contacted by two SC electrodes on both sides presents pronounced oscillations of the critical current $I_{c}$ as a function of the magnetic field $B_{\|}$ applied parallel to the nanowire axis, resulting in an $I_{c}\left(B_{\|}\right)$ behavior akin to a SQUID. The period in $B_{\|}$ corresponds to the magnetic flux $\Phi_{0}=h/2e$ , the SC flux quantum, threading the TI nanowire. These results strongly suggest that the side-contacted TI-nanowire junction forms an intrinsic nano-SQUID, where the top and bottom surfaces of the nanowire effectively work as two parallel SC-normal-SC (SNS) junctions, while the bulk is insulating; the threading magnetic flux Φ imposes an SC phase difference between the two SNS junctions, leading to the $I_{c}$ oscillations. Therefore, it is interesting to determine if such a nano-SQUID formed intrinsically in a TI-nanowire junction would present a Josephson diode effect, as in other asymmetric SQUIDs (*11*–*13*). If it does, such a diode has the potential advantage of being highly tunable by both the parallel magnetic field $B_{∥}$ and the back-gate voltage $V_{G}$ , which introduce time-reversal and inversion symmetry breaking, respectively.

Here, we demonstrate that the side-contacted TI-nanowire junction indeed presents a large and tunable Josephson diode effect. The diode efficiency η changes sign with both $B_{∥}$ and the back-gate voltage $V_{G}$ , and its magnitude reaches 0.3, which is comparable to the largest reported for single Josephson junctions (*16*) without vortex trapping. Our devices are based on TI nanowires with 1 to 2 μm in length, 10 to 20 nm in thickness, and 60 to 80 nm in width, dry-etched from an exfoliated flake of the bulk-insulating TI material BiSbTeSe_2_ and side-contacted by Nb electrodes. This structure allows for efficient back-gating, which tunes the asymmetry between the two SQUID arms, an essential ingredient of the Josephson diode effect in the SQUID configuration (*12*). The interface between the TI and Nb has been reported to be sufficiently transparent to give rise to a highly skewed current-phase relationship (*25*), which is also crucial for the Josephson diode effect (*12*); the spin-momentum locking in the TI surface state guarantees a perfectly transmitted Andreev mode (*26*, *27*) and enhances the skewness (*25*). Our full three-dimensional (3D) simulations of the TI nanowire side-contacted by a conventional superconductor reproduce all the key features observed experimentally, giving confidence in the nano-SQUID origin of the Josephson diode effect. Moreover, our theoretical analysis shows that the sign change in η corresponds to a topological phase transition in the TI nanowire, which should be accompanied by the emergence of Majorana zero modes (*24*). Hence, the relatively simple platform reported here is interesting not only for future SC electronics but also for Majorana physics.

## RESULTS

### Josephson diode effect in a side-contacted TI nanowire junction

**Fig. 1.**
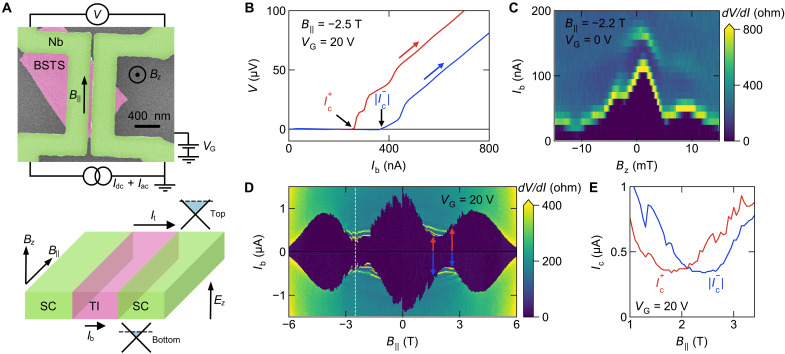
Device schematics and the behavior of the critical current. (**A**) False-color SEM image of device A with schematics of the pseudo-four-terminal measurement. An exfoliated TI flake of BiSbTeSe_2_ (BSTS) is dry-etched into a nanowire (pink). The etched area is filled with Nb (green) to form a sandwich junction. There are remaining TI pieces that do not play any role. The lower drawing shows that the supercurrents flow through the top and bottom topological surface states, whose chemical potentials can be made different by a back-gate creating the electric field $E_{z}$ ; a magnetic field applied parallel to the nanowire axis causes the Josephson diode effect. (**B**) *I*-*V* characteristics of device A taken at $B_{\|}=-2.5$ T and $V_{G}=20$ V. For better comparability with the positive bias curve (red), the negative bias curve (blue) was flipped horizontally and vertically. The current sweep was always from zero as indicated by the red and blue arrows. (**C**) Color map of dV/dI as function of out-of-plane magnetic field $B_{z}$ and $I_{b}$ at $B_{\|}=2.2$ T and $V_{G}=0$ V in device A. (**D**) Color map of dV/dI as function of $B_{\|}$ and $I_{b}$ at $V_{G}=20$ V in device A. Red and blue arrows highlight the diode effect. The white dashed line mark the $B_{\|}$ value where the data in (B) was taken. (**E**) Plot of $I_{c}^{+}$ and $|I_{c}^{-}|$ extracted from the data in (D) as function of $B_{\|}$ near an $I_{c}$ minimum at $B_{\|}\approx 2$ T; here, the critical currents are approximated by the $I_{b}$ value at which dV/dI exceeded 50 ohms.

Figure 1A shows a false-color scanning electron microscope (SEM) image of our device A, including the schematics of the pseudo-four-terminal measurement. The Nb electrodes had the transition temperature $T_{c}\approx 7$ K, and the in-plane upper critical field $H_{c2,∥}$ was slightly above 6 T, which was the highest magnetic field available in this experiment. As described in (*24*), the precise alignment of the parallel magnetic field $B_{∥}$ using a vector magnet was crucial for this type of device. Even a small $B_{z}$ component will cause the ordinary Fraunhofer interference effect and reduce the $I_{c}$ , messing up the $I_{c}\left(B_{∥}\right)$ behavior. We show in Fig. 1C an example of the Fraunhofer pattern measured as a function of $B_{z}$ in the presence of $B_{∥}=2.2$ T, where one can see that $B_{z}$ of just a few millitesla reduces $I_{c}$ substantially [the asymmetry in the pattern in Fig. 1C is due to an anomalous phase created by $B_{\|}$ (*28*), which does not open a gap but shifts the Fermi surface in the momentum space (*29*)]. In the following, we focus on results obtained from device A (shown in Fig. 1A), and additional data from three more devices are shown in the Supplementary Materials. Unless otherwise noted, the data were acquired at the base temperature of our dry dilution refrigerator (≈30 mK).

In Fig. 1B, we show the current-voltage (*I*-*V*) characteristics measured at $B_{\|}=-2.5$ T and $V_{G}=20$ V to present the Josephson diode effect: The data for positive current $I^{+}$ are shown in red, while the data for negative current $I^{-}$ are shown in blue and are plotted for $|\;I^{-}\;|$ with flipped voltage. In these measurements, we have always swept the current from zero to avoid hysteresis or other complications. The data in Fig. 1B show the critical currents $I_{c}^{+}=250$ nA and $|\;I_{c}^{-}\;|=390$ nA, giving the diode efficiency $\eta \equiv \left(I_{c}^{+}-|I_{c}^{-}|\right)/\left(I_{c}^{+}+|\;I_{c}^{-}|\right)\approx -0.23$. Figure 1D maps the differential resistance dV/dI measured across the junction as a function of $B_{\|}$ and the bias current $I_{b}$ taken at $V_{G}=20$ V; the dark area corresponds to zero resistance and its boundary marks $I_{c}$ . The pronounced oscillations of $I_{c}$ as a function of $B_{\|}$ having the periodicity of $\Phi_{0}$ ( =h/2e ) are due to the intrinsic nano-SQUID formed in our junction (*24*).

One can see in Fig. 1D that for most of the $B_{\|}$ values, $|\;I_{c}^{+}|$ and $|\;I_{c}^{-}|$ are not the same. This is particularly clear in the region near the $I_{c}$ minima (e.g., around $B_{\|}\approx 2$ T). Across the $I_{c}$ minima, the sign of η changes, as highlighted by red and blue arrows. This sign change is more clearly demonstrated in Fig. 1E, where the $B_{\|}$ dependencies of $I_{c}^{+}$ and $|\;I_{c}^{-}|$ are plotted in red and blue, respectively. The crossing of $I_{c}^{+}\left(B\right)$ and $|I_{c}^{-}\left(B\right)\;|$ at $B_{\|}\approx 2$ T corresponds to the sign change in η.

### Gate tunability of the Josephson diode effect

**Fig. 2.**
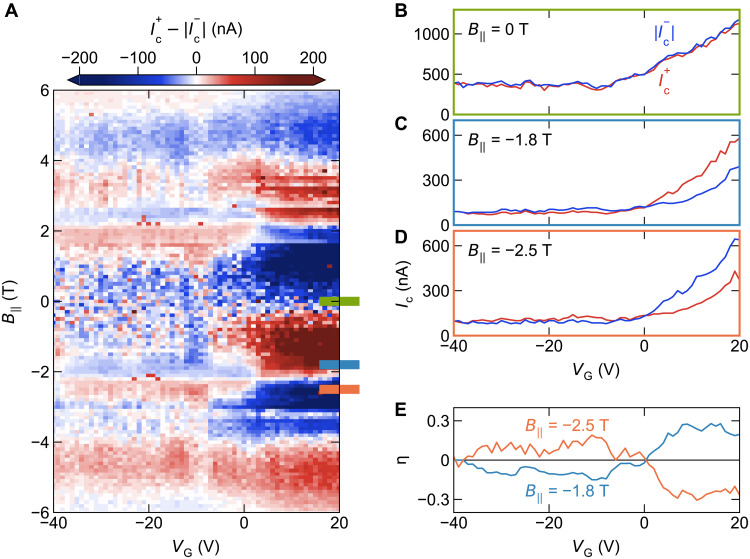
Tunability of the diode efficiency. (**A**) Color mapping of $I_{c}^{+}-|I_{c}^{-}|$ as a function of $V_{G}$ and $B_{\|}$ in device A, demonstrating the tunability of the Josephson diode effect. (**B** to **D**) Raw data of $I_{c}^{+}\left(V_{G}\right)$ and $|I_{c}^{-}\left(V_{G}\right)|$ used for producing (A) at $B_{\|}=0$ , −1.8 , and −2.5 T; these $B_{\|}$ values are marked in (A) with corresponding colors. (**E**) $V_{G}$ dependence of the diode efficiency η for $B_{\|}=-1.8$ and −2.5 T calculated from the data in (C) and (D).

In our TI-nanowire junction, one can tune the sign and the magnitude of the diode effect not only with $B_{\|}$ but also with $V_{G}$ . The gate tunability of $I_{c}$ in zero field is shown in Fig. 2B, where $I_{c}$ changes roughly by a factor of three with $V_{G}$ . In Fig. 2A, we plot $I_{c}^{+}-|I_{c}^{-}|$ as a function of $B_{\|}$ and $V_{G}$ as a color map, where the white color corresponds to zero (i.e., no diode effect). One can see that the diode effect oscillates with $B_{\|}$ , resulting in alternation of red and blue; the magnitude of $I_{c}^{+}-|I_{c}^{-}|$ can exceed 200 nA at positive $V_{G}$ , which is large since $|I_{c}|≲1$ μA at all times (see Fig. 1D). We notice that $I_{c}^{+}-|I_{c}^{-}|$ is essentially antisymmetric in $B_{\|}$ , as expected.

**Fig. 3.**
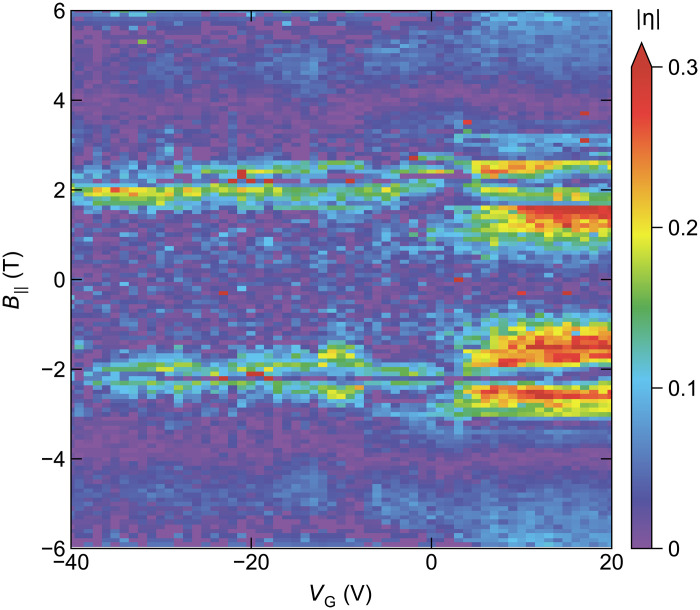
Magnitude of the diode efficiency. Color mapping of ∣η∣ as a function of $V_{G}$ and $B_{\|}$ in device A, showing that the magnitude of the diode efficiency can reach 0.3 near the sign-switching magnetic field $B_{\|}\approx \pm 2$ T.

We observed that for $|B_{\|}\;|≲3$ T and at $|B_{\|}|\approx 6$ T, the nonreciprocity $I_{c}^{+}-|I_{c}^{-}|$ presents a gate-induced sign change. To make this behavior clear, we plot the $V_{G}$ dependencies of $I_{c}^{+}$ and $|I_{c}^{-}|$ in red and blue lines, respectively, for $B_{\|}=0$ , −1.8 , and −2.5 T in Fig. 2 (B to D). One can see that the two lines cross at −5 V $≲V_{G}≲$ 0 V in $B_{\|}=-1.8$ and −2.5 T, while they are essentially indistinguishable at 0 T. One can also notice that the way the sign change occurs is opposite between $B_{\|}=-1.8$ and −2.5 T; namely, η ( $\propto I_{c}^{+}-|I_{c}^{-}|$ ) changes from negative to positive with increasing $V_{G}$ in $B_{\|}=-1.8$ T, while it is opposite in $B_{\|}=-2.5$ T. To further visualize the $V_{G}$-induced sign change, in Fig. 2E, we plot η in $B_{\|}=-1.8$ and −2.5 T as a function of $V_{G}$ , where the peculiar sign-changing behavior is apparent. We note that ∣η∣ reaches 0.3, which is large (*2*, *12*–*14*, *23*, *30*). Figure 3 depicts the parameter regime where this large diode effect is observed; one can see that ∣η∣≈0.3 is achieved near $B_{\|}\approx \pm 2$ T where the sign change of ∣η∣ takes place.

**Fig. 4.**
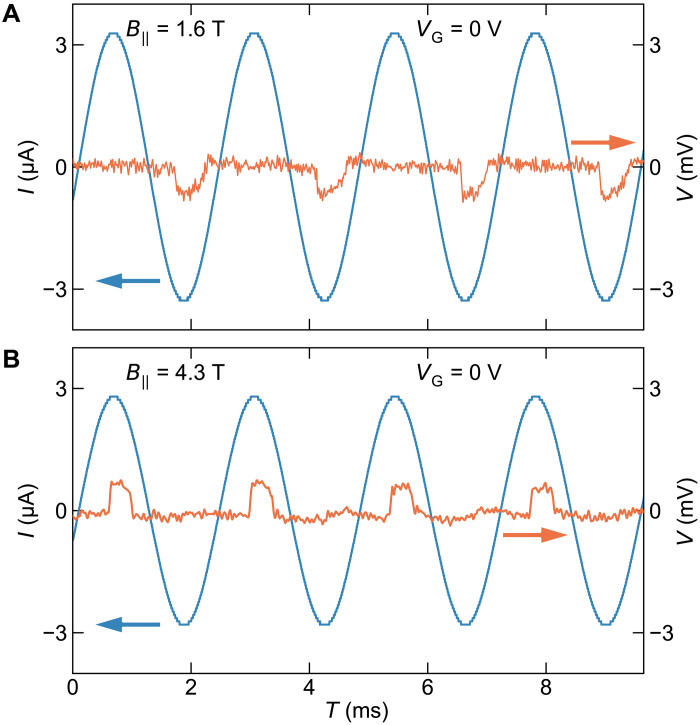
Visualization of the rectification effect. (**A** and **B**) Time-resolved measurement of the voltage drop across the Josephson junction (orange curve) in response to a sinusoidal ac with an amplitude of ~3 μA (blue), highlighting the rectification effect. The data in (A) and (B) were acquired at $B_{\|}=1.6$ and 4.3 T, respectively, where the sign of η was opposite. The measurement was performed on device B.

To demonstrate the rectification behavior resulting from our Josephson diode, we performed simple time-domain measurements using another junction, device B: We recorded the voltage waveform when a simple sinusoidal ac was applied to the junction. In $B_{\|}=1.6$ T, as shown in Fig. 4A, when the ac amplitude is ∼3 μA (which is larger than $|I_{c}^{-}|$ but smaller than $|I_{c}^{+}|$ ), the junction presents a rectified waveform with only negative voltage appearing for negative current. In $B_{\|}=4.3$ T, on the other hand, the sign change in η results in the opposite rectification as shown in Fig. 4B. This experiment demonstrates that the diode polarity is easily switched by the magnetic field. One can similarly switch the diode polarity with gate voltage, as shown in Fig. 2E. The ac response in Fig. 4 is limited by the low-pass filters in the signal line; it would be interesting to elucidate the intrinsic frequency limit of this type of SC diode.

### Theoretical analysis and simulations

The behavior of the Josephson junction can be modeled phenomenologically as a nano-SQUID, where the supercurrent is carried separately by the top and bottom surfaces with skewed current-phase relations

$$I_{\rho }\left(\theta_{\rho }\right)=I_{0,\rho }\left[\text{sin}\left(\theta_{\rho }\right)+S_{\rho }\text{sin}\left(2\theta_{\rho }\right)\right]\;$$ (1)

where $I_{0,\rho }$ and $S_{\rho }$ determine the magnitude and the skewness of the current-phase relation, and $\theta_{\rho }$ is the SC phase difference at the top and bottom surfaces ( ρ=t,b ). The total Josephson current is $I\left(\theta \right)=\sum_{\rho =t,b}I_{\rho }\left(\theta_{\rho }\right)$ . The magnetic flux Φ threading through the TI nanowire dictates $\theta_{t}-\theta_{b}=2\pi \frac{\Phi }{\Phi_{0}}\equiv \phi$ . The area that determines Φ in the nano-SQUID model is affected by the localization depth of the TI surface states below the nominal surface (*24*), as well as the London penetration depth of the superconductor. The sign of $S_{\rho }$ determines the skewness direction. Skewed current-phase relation arise naturally in high-transparency Josephson junctions and are right skewed ( $S_{\rho }<0$ ) (*31*, *32*), and intermediate transparency leads to $|\;S_{\rho }\;|\ll 1$ ; a direct measurement of the current-phase relation for the same TI material was reported in (*25*), which found a typical skewness $|\;S_{\rho }\;|\approx 0.2$.

A Josephson diode effect in our TI-based nano-SQUID occurs under the following conditions: (i) A magnetic flux through the nanowire breaking time-reversal symmetry; (ii) an asymmetry between the top and bottom surfaces, $I_{0,t}\neq I_{0,b}$ and/or $S_{t}\neq S_{b}$ , which breaks inversion symmetry (*33*–*35*); and (iii) a finite skewness of the current-phase relation $|\;S_{\rho }\;|>0$ . Time-reversal and inversion symmetry must be broken because their action reverts the direction of current flow. The gate-induced top/bottom asymmetry is enough for the present diode effect, and there is no need for bulk nor structure inversion asymmetry. The skewness $|\;S_{\rho }\;|>0$ ensures the presence of higher harmonics in the current-phase relation that are necessary for the critical currents in opposite directions to be distinct. Conversely, for zero skewness, both individual and total current-phase relations are sinusoidal so that, in this case, the critical currents in both directions are equal. While the nonreciprocal transport and an SC diode effect have both been discussed for TI nanowires with the current flowing along the wire axis (*34*, *35*), in the present nano-SQUID setup, the current runs perpendicular to the wire axis.

**Fig. 5.**
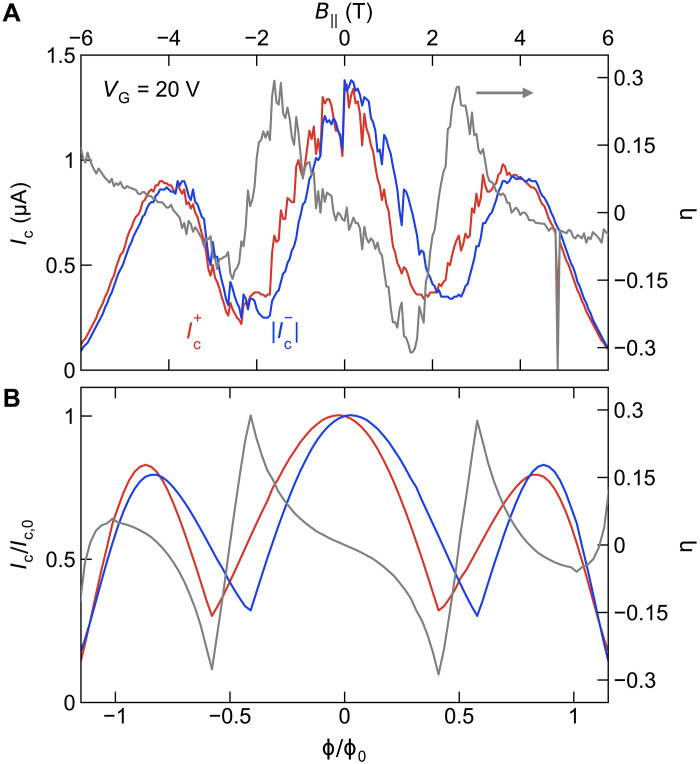
Comparison with a theoretical simulation. (**A**) Experimental data of the critical currents $I_{c}^{+}$ and $|I_{c}^{-}|$ as a function of parallel magnetic field, together with the diode efficiency as measured in device A. (**B**) Simulation based on a tight-binding model of a TI nanowire proximitized from both sides by a conventional superconductor. This simulation result corresponds to the case of $S_{t}=-0.2$ , $S_{b}=-0.2$ , and $I_{0,t}/I_{0,b}$ = 0.8 in the phenomenological model, Eq. 1.

We further performed a full 3D numerical simulation of a TI-nanowire Josephson junction using a tight-binding model (details presented in Materials and Methods). The comparison between the simulation results and the relevant experimental data for the critical currents $I_{c}^{+}$ and $|\;I_{c}^{-}|$ , together with the diode efficiency, is shown in Fig. 5. As the chemical potential is placed within the bulk bandgap of the 3D TI, the current is carried only through the top and bottom surfaces of the nanowire, which yields the nano-SQUID picture. The simulation faithfully reproduces the behavior of critical currents as well as the magnitude of the diode effect, exhibiting sign changes of η at $\Phi =\pm \Phi_{0}/2$ . Crucially, the diode effect appears in the calculation only when the asymmetry between the top and bottom surfaces is present.

In the nano-SQUID model, time-reversal symmetry is restored at integer multiples of half a flux quantum, $\Phi =\frac{n}{2}\Phi_{0},\;n\in \mathbb{Z}$ . As a consequence, the critical current asymmetry must flip when the flux is mirrored around each half flux quantum, $I_{c\pm }\left(\frac{n}{2}\Phi_{0}+\delta \Phi \right)=-I_{c\mp }\left(\frac{n}{2}\Phi_{0}-\delta \Phi \right)$ . This requires the diode efficiency η to be periodic and odd around each $\Phi =\frac{n}{2}\Phi_{0}$ . This behavior is approximately observed in the microscopic simulation and in the experiments. The deviation from the expected periodicity near $\Phi /\Phi_{0}=\pm 1$ is due to additional effects such as the suppression of superconductivity in the Nb leads and Zeeman coupling. To complement this microscopic simulation, we also calculated the behavior of the diode effect from the phenomenological nano-SQUID model (Eq. 1), reproducing the key features of the experimental data, as shown in fig. S7.

In a closed system at equilibrium, the SC phase difference across the junction must satisfy the condition that current is conserved

$$I_{t}\left(\theta_{0}+\phi \right)=-I_{b}\left(\theta_{0}\right)\;$$

Here, a gauge is chosen to include the effect of the magnetic flux in the phase difference of the top junction (i.e., $\phi =2\pi \Phi /\Phi_{0}$ ). This equation has two or more solutions due to the periodicity of the supercurrent with the SC phase difference, but the system selects the solution that minimizes the free energy of the junction, fixing the equilibrium phase bias $\theta_{0}$ . At zero temperature, the free energy is given by the Josephson energy $E_{J}=\frac{\hbar }{2e}\int^{\theta }d\theta^{\prime }I\left(\theta^{\prime }\right)$ . As the flux is increased from Φ=0 to $\Phi_{0}$ , the gauge-invariant phase difference $\gamma_{t,b}=\theta_{t,b}+e\int Adl/\hbar$ on the weaker junction winds from 0 to 2π while the phase difference of the other junction remains close to 0. For small skewness or large asymmetry, this phase winding is continuous. For larger skewness above a critical value set by the asymmetry, the equilibrium phase difference jumps discontinuously by Δθ at the flux bias $\Phi =\Phi_{0}/2$ , changing from $\theta_{0}$ to $\theta_{0}+\Delta \theta$ with Δθ≈π . This first-order transition is expected for typical skewness ( $|S_{\rho }|\approx 0.2$ ) of our device. We confirmed this result from calculations for the phenomenological nano-SQUID model, as shown in fig. S7.

## DISCUSSION

Now, we discuss the relevance of topology in the present experiment. Since our TI-nanowire junction effectively forms a nano-SQUID consisting of two SNS junctions at the top and bottom TI surfaces, each SNS junction can be in the topological phase with odd fermion parity in its ground state when the phase difference is between π and 3π (mod 4π ) (*36*). Since we have two SNS junctions, the TI-nanowire junction as a whole is in the topological phase when only one of the two SNS junctions is topological (*24*); in this case, Majorana zero modes are expected to show up at the ends of the TI nanowire. It was theoretically shown in (*24*) that when the two junctions are asymmetric, this topological phase is realized in equilibrium in the magnetic-flux range of $\left(n-\frac{1}{2}\right)\Phi_{0}<\Phi <\left(n+\frac{1}{2}\right)\Phi_{0}$ with odd-integer n.

In this nano-SQUID, both the diode sign reversal and the topological phase transition are pinned to half flux quanta by a common origin—the restoration of time-reversal symmetry at these magnetic fluxes. Time-reversal symmetry pins the phase differences of the top and bottom Josephson junctions to 0 or π , where the latter is the topological phase transition point of a single junction (*36*). The asymmetry between the top and bottom junctions can be controlled by the gate voltage $V_{G}$ in our setup; the junction having the smaller Josephson energy will experience the phase winding, thereby undergoing the topological transition. Upon exchanging the top-bottom asymmetry by gating, the junction that experiences the 0–π transition switches, which coincides with the gate-induced sign reversal of the diode effect.

In the experiments to detect the Josephson diode effect, a bias current is passed through the junction, and it dictates the phase difference across the junction according to the current-phase relation. This means that the phase is not a free parameter in the current-biased experiment and the persistence of the topological phase is not guaranteed upon current biasing. It is an interesting topic of future research to clarify the role of current bias to control the topological phase transition.

In conclusion, we found that the intrinsic nano-SQUID nature of the TI-nanowire junction (*24*) leads to a large Josephson diode effect, whose size and sign can be tuned by the parallel magnetic field $B_{\|}$ and the back-gate voltage $V_{G}$ . Our theoretical modeling shows that the sign change in the Josephson diode efficiency η as a function of $B_{\|}$ is accompanied by a topological phase transition in equilibrium, making the TI-nanowire junction interesting not only for SC electronics but also for studying topological superconductivity and Majorana zero modes. Moreover, given that other tunable Josephson diodes based on SQUID geometry tend to be large and complicated (*12*, *13*), the small and simple nature of our device is an advantage for integrating the Josephson diodes in large-scale circuits, although the necessity of a magnetic field can be a limiting factor.

## MATERIALS AND METHODS

### Materials and device fabrications

Bulk single crystals of BiSbTeSe_2_ were grown using the modified Bridgman method, following the procedure outlined in (*37*), with high-purity (99.9999%) Bi, Sb, Te, and Se as starting materials. Thin flakes of BiSbTeSe_2_ were mechanically exfoliated from a bulk crystal and transferred onto a degenerately doped Si wafer coated with a 290-nm SiO_2_ layer, which acts as a dielectric for the back gate. Flakes suitable for device fabrication were identified under an optical microscope. Nanowires and Josephson junctions were patterned using electron beam lithography, with ZEP520A resist exposed via a Raith PIONEER Two system. The BiSbTeSe_2_ flakes were dry-etched into nanowires using Ar plasma. Subsequently, 45 nm of Nb was sputter-deposited to form the junctions. The device geometry was precisely characterized using SEM and atomic force microscopy after the completion of measurements.

### Measurements

Transport measurements were conducted at the base temperature (~30 mK) of our Oxford Instruments TRITON 300 dry dilution refrigerator. To minimize noise, we equipped the electrical lines with RC and copper-powder filters. We used a quasi-four-probe configuration to measure the differential resistance dV/dI across the Josephson junction using a standard low-frequency lock-in technique, where a small ac of 5 nA was superimposed on a dc bias. Whenever a four-terminal measurement was not possible due to defective leads, a quasi-three-probe technique was applied instead (which was the case for device A). To apply magnetic fields, we used a 6/1/1-T SC vector magnet.

### Simulations

To simulate the behavior of our devices, we use a 3D lattice model with two SC side electrodes and a central TI region. The TI component of the device is described using a standard phenomenological 3D massive Dirac fermion model. The Hamiltonian of the model is given by

$$\begin{array}{c} H_{\text{TI}}\left(k\right)=A_{1}k_{\|}s_{z}\sigma_{x}+A_{2}\left(k_{x}s_{x}+k_{z}s_{y}\right)\sigma_{x}\\ \;+\left[M-B_{1}k_{\|}^{2}-B_{2}\left(k_{x}^{2}+k_{z}^{2}\right)\right]s_{0}\sigma_{z}\\ \;+\left[C+D_{1}k_{\|}^{2}+D_{2}\left(k_{x}^{2}+k_{z}^{2}\right)-\mu_{\text{TI}}\right]s_{0}\sigma_{0} \end{array}$$ (2)

where $s_{i}$ and $\sigma_{i}$ are Pauli matrices describing spin and orbital degrees of freedom, respectively. The niobium superconductor that forms the Josephson junction is described using a simple parabolic band with the Hamiltonian

$$H_{\text{SC}}\left(k\right)=\left[t\left(k_{x}^{2}+k_{\|}^{2}+k_{z}^{2}\right)-\mu_{\text{SC}}\right]s_{0}\sigma_{0}$$ (3)

Both Hamiltonians are discretized on a cubic lattice with the spacing $a_{\text{lat}}=1$ nm using the finite difference method with nearest-neighbor hoppings. Since the devices under study are much longer ( L> 1 μm) than their width or height ( W,H ; order of 10 nm), it is assumed that translational invariance is retained in direction parallel to the nanowire, and thus, $k_{\|}$ remains a good quantum number. After the discretization, both systems are combined in a single real-space Hamiltonian, which is given by

$$H_{0}\left(x\right)=H_{\text{SC}}\theta \left(-x\right)+H_{\text{TI}}\left[\theta \left(x\right)-\theta \left(W-x\right)\right]+H_{\text{SC}}\theta \left(x-W\right)$$ (4)

where W is the width of the TI nanowire and θ(x) is the Heaviside step function. The matrix representation of this real-space Hamiltonian was constructed using Kwant package (*38*). We include the effect of magnetic field via the Peierls substitution, where the hoppings between sites $r_{n}$ and $r_{m}$ are replaced by

$$t_{\mathrm{mn}}\to t_{\mathrm{mn}}\text{exp}\left[-i\tau_{z}\frac{e}{\hbar }\int_{r_{n}}^{r_{m}}dr\cdot A\left(r\right)\right]$$ (5)

where $\tau_{z}=\pm 1$ for particle and hole degrees of freedom, respectively. In all the calculations, we use the gauge where $A\left(r\right)=-B_{\|}z\hat{x}$ . This assumes that the magnetic field penetrates the device fully, which is justified as the thickness of the SC electrodes (about 45 nm) is comparable to the London penetration depth of niobium. The flux through the junction is then established as $\Phi =B_{\|}\tilde{A}$ , where $\tilde{A}$ is the effective junction area.

To enable the SC diode effect, asymmetry between the top and bottom surface state of the nanowire has to be introduced through the gate electrostatic potential, which we model by a gradient of the chemical potential inside of the TI, $\mu_{\text{TI}}\left(z\right)=\mu_{\text{TI}}+V_{\mathrm{GT}}z/H$ . The superconductivity then is treated using Bogoliubov–de Gennes (BdG) Hamiltonian

$$H_{\text{BdG}}=\left[\begin{array}{cc} H_{0}\left(k\right) & \Delta \\ \Delta^{†} & -H_{0}^{T}\left(-k\right) \end{array}\right]$$ (6)

where the SC order parameter Δ is

$$\Delta \left(x\right)=\Delta_{0}\left[\text{exp}\left(-i\phi /2\right)\theta \left(-x\right)+\text{exp}\left(i\phi /2\right)\theta \left(x-W\right)\right]{\text{is}}_{y}\sigma_{0}$$ (7)

with ϕ being the phase difference between the SC condensates. With such a setup constructed, we then calculate the current-phase relations for varying magnetic field by using the excitation spectrum of the BdG Hamiltonian. The Josephson current density across the quasi-1D nanowire is expressed through the positive eigenvalues that are a function of the momentum component parallel to the nanowire axis

$$j\left(\phi \right)=-\frac{e}{\hbar }\sum_{\epsilon_{n}>0}\int \frac{{\mathrm{dk}}_{\|}}{2\pi }\text{tanh}\left[\frac{\epsilon_{n}\left(k_{\|}\right)}{2k_{B}T}\right]\frac{d\epsilon_{n}\left(k_{\|}\right)}{d\phi }$$ (8)

where $k_{B}$ is the Boltzmann constant and T is the temperature. The integration is performed by discretizing momenta and calculating the spectrum at 281 points for $k_{\|}a_{\text{lat}}$ in the range [−1.4, 1.4]. From the supercurrent density, the total current through the junction is obtained as I(ϕ)=j(ϕ)L , and then, the critical supercurrent in both directions is determined as $I_{c+}=\text{max}\left[I\left(\phi \right)\right]$ and $I_{c-}=\text{min}\left[I\left(\phi \right)\right]$ , respectively. In the simulations, the parameters are as follows: $a_{\text{lat}}=1$ , $A_{1}=0.25$ , $A_{2}=0.35$ , $B_{1}=-0.3$ , $B_{2}=-0.6$ , C=0 , $D_{1}=0.025$ , $D_{2}=-0.05$ , M=−0.25 , $\mu_{\text{TI}}=0.0$ , $V_{\mathrm{GT}}=0.05$ , t=1.0 , $\mu_{\text{SC}}=1.0$ , $\Delta_{0}=0.08$ , W=14 , $W_{\text{SC}}=16$ , H=15 , and L=1500 , with all the energies expressed in electron volts and all the lengths in nanometers. The phenomenological model parameters are chosen such that the bulk gap of the TI is in agreement with the experimentally determined gap of 250 to 300 meV of BiSbSeTe_2_. These parameters also translate to a topological surface state with a Fermi velocity $v_{F}\approx 4\times 10^{5}\;$m/s. Since the chemical potential changes between the top and the bottom surfaces of the TI, the Fermi wave length changes as well, but generally, it is between 25 and 65 nm. The imperfect transmission at the superconductor 3D TI interface was modeled by the Fermi wave vector mismatch, which was $k_{F,\text{SC}}/k_{F,\text{TI}}\approx 4$ . To make the simulations numerically tractable, the use of lattice discretization with 1-nm spacing required increasing the SC gap magnitude from the experimental value of $\Delta_{\text{exp}}\approx 0.9$ meV. The TI-nanowire width is chosen so that the minigap of the calculated spectrum of states in the junction is equal to about 40 meV, which is approximately ½ of the gap of the SC electrodes in the model ( $\Delta_{0}=80$ meV). This fraction was chosen to be consistent with the size of the induced gap measured in the studied device. One can also estimate the fictitious SC coherence length in the simulation to be $\xi =\hbar v_{F}/\Delta_{0}=3.3\;$ nm using $v_{F}$ of the TI surface state and the model SC gap in the leads. The $V_{\mathrm{GT}}$ value used for the simulation in Fig. 5B is chosen because it produces the largest diode efficiency.
