# Supplementary material for: Tunable superconducting diode effect in a topological nano-SQUID
Source: Sci Adv. 2025 Sep 19;11(38):eadw4898. doi: 10.1126/sciadv.adw4898 (PMC12448122; doi:10.1126/sciadv.adw4898)
Supplement: Supplementary file 1 — Supplementary Text Figs. S1 to S8 Table S1 [file sciadv.adw4898_sm.pdf]

Science Advances
AAAS

Supplementary Materials for

Tunable superconducting diode effect in a topological nano-SQUID

Ella Nikodem *et al.*

Corresponding author: Yoichi Ando, ando@ph2.uni-koeln.de

Sci. Adv. **11**, eadw4898 (2025)
DOI: 10.1126/sciadv.adw4898

This PDF file includes:

Supplementary Text
Figs. S1 to S8
Table S1

Supplementary Text

Experimental details and additional data

Sample geometry

Following the transport measurements, the device geometry was accurately characterized using the scanning electron microscopy (SEM) images shown in Figs. S1A-D to determine the length L and width W of the nanowires. The nanowire thickness was measured using atomic force microscopy (AFM). The geometric parameters for devices A, B, C, and D are summarized in Table S1.

Magnetic field alignment

The precise alignment of the magnetic field relative to the junction's coordinate system is crucial for studying the junction properties in magnetic fields parallel to the nanowire axis, $B_{||}$. Misalignment introduces a nonzero B_z component, leading to phase winding and a current modulation along the junction, as observed in a Fraunhofer measurement. To achieve good alignment, multiple Fraunhofer-type measurements were performed with intentionally-applied small B_z fields in the presence of large nominal $B_{||}$ fields. These data reveal a systematic shift in the position of the maximum critical current I_c arising from a spurious B_z component caused by misalignment. By identifying the necessary B_z value to compensate the spurious B_z component for each nominal $B_{||}$, we achieved the necessary alignment of $B_{||}$. The precise procedure is described in Ref. [24].

On the other hand, a small misalignment of the magnetic field within the device plane from the nanowire axis does not affect the data in any noticeable manner.

Reproducibility of the Josephson diode effect

In Figs. S2, S3, S4, we show data for three more devices B, C, and D not presented in the main text. As shown in Fig. S1, the device design is essentially the same as the one discussed in the main text. All key features are reproduced in these devices. In the presence of a finite $B_{||}$, all of the devices develop a pronounced Josephson diode effect that is periodic in $B_{||}$. Note that in contrast to devices C and D, device B was not gate-tunable due to an accidental short to the back gate. Intriguingly, in device D we observe two sign changes of the diode efficiency as a function of V_{G} as shown in Fig.

S4D.

Josephson diode effect at 1 K

Figures S5 and S6 show $I_c^+ - |I_c^-|$ and the diode efficiency $|\eta|$, respectively, as a function of V_G and $B_{||}$ for devices A, C, and D measured at $T = 1$ K. The tunability of $I_c^+ - |I_c^-|$ remains unaffected by the increased temperature, while the magnitude of I_c in both bias directions is slightly reduced. The diode efficiency is largely robust against the temperature increase.

Mechanism of the sign reversal of the diode effect

The phenomenological nano-SQUID model, Eq. (1) in the main text, predicts a sign reversal of the diode efficiency at integer multiples of half a magnetic flux quantum, see Fig. S7 for results derived from the nano-SQUID model. This is a robust feature of the nano-SQUID description because it is tied to the restoration of time-reversal symmetry at these flux values. Notably, the sign reversal of the diode efficiency is also observed in the experimental data (Fig. 2A in the main text) for the whole range of gate voltages at the magnetic field corresponding to the flux $\Phi = \Phi_0/2$. Qualitative agreement of the experimental data with the phenomenological model supports the conclusion that the supercurrent is predominantly carried by the top and bottom surface states separately. Further, we compute the diode efficiency as a function of top-bottom asymmetry $\frac{I_{0,t}-I_{0,b}}{I_{0,t}+I_{0,b}}$ and magnetic flux, with the result shown in Fig. S8. The diode efficiency changes sign around the time-reversal symmetry points $\Phi = \frac{n}{2}\Phi_0$, $n \in \mathbb{Z}$ as well as when the top-bottom asymmetry is exchanged. Already with fixed skewness $S_t = S_b = -0.2$, the diode efficiency approaches the theoretical maximum of $|\eta| = \frac{1}{3}$ that is achievable with current-phase relations containing only first- and second harmonic components. (The theoretical maximum $|\eta| = \frac{1}{3}$ for current-phase relations with first- and second-harmonic components of the form $I_0\,(\sin(\theta) + S\sin(2\theta + \delta\theta))$ is reached for $S = 0.5$ and $\delta\theta = \pi/2$.)

The sign reversal of the diode effect as a function of the gate voltage V_G also appears near $V_G \approx 0$ in the experimental data (Fig. 2E) for several magnetic field values. The direction of the diode effect can thus be controlled by the gate voltage V_G, in addition to the magnetic flux that threads the nanowire. Furthermore, for a large positive gate voltage V_G, the experimental diode efficiency approaches the theoretical maximum of $|\eta| = \frac{1}{3}$ mentioned above. While the experimental current-

phase relations may contain any order of harmonics, we expect that the second harmonic dominates over the higher harmonics because the long nanowire has many transverse modes for which higher harmonics are suppressed by specular reflection competing with the Andreev reflection at the superconductor interface. This picture is also confirmed by our microscopic simulations, where we observed $|\eta| < 1/3$.

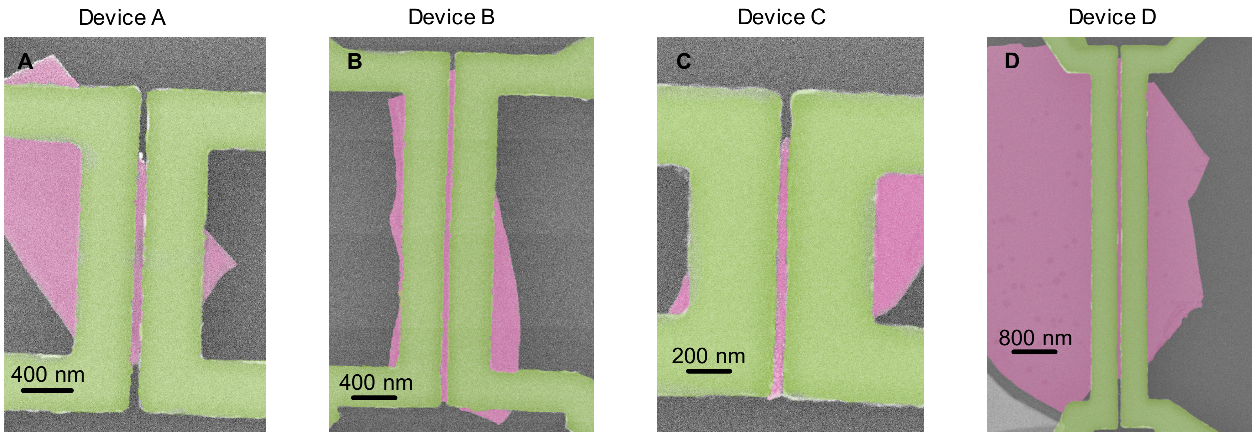

Figure S1: **SEM images of devices A-D.**

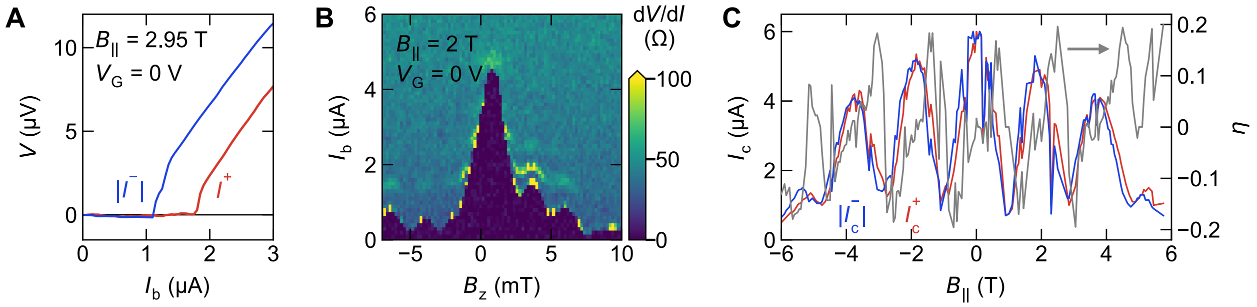

Figure S2: **Reproducibility in device B. A,** I-V characteristics at $B_\parallel = 2.95$ T and $V_G = 0$ V. The negative bias curve (blue) was flipped horizontally and vertically. The current sweep was always from zero. **B,** Color map of dV/dI as a function of B_z and I_b at $B_\parallel = 2$ T and $V_G = 0$ V. **C,** I_c for positive bias currents (red) and negative bias currents (blue), as well as the diode efficiency η (grey) as a function of $B_\parallel$ at $V_G = 0$ V. This device was not gate-tunable. Measurements were at $T = 30$ mK.

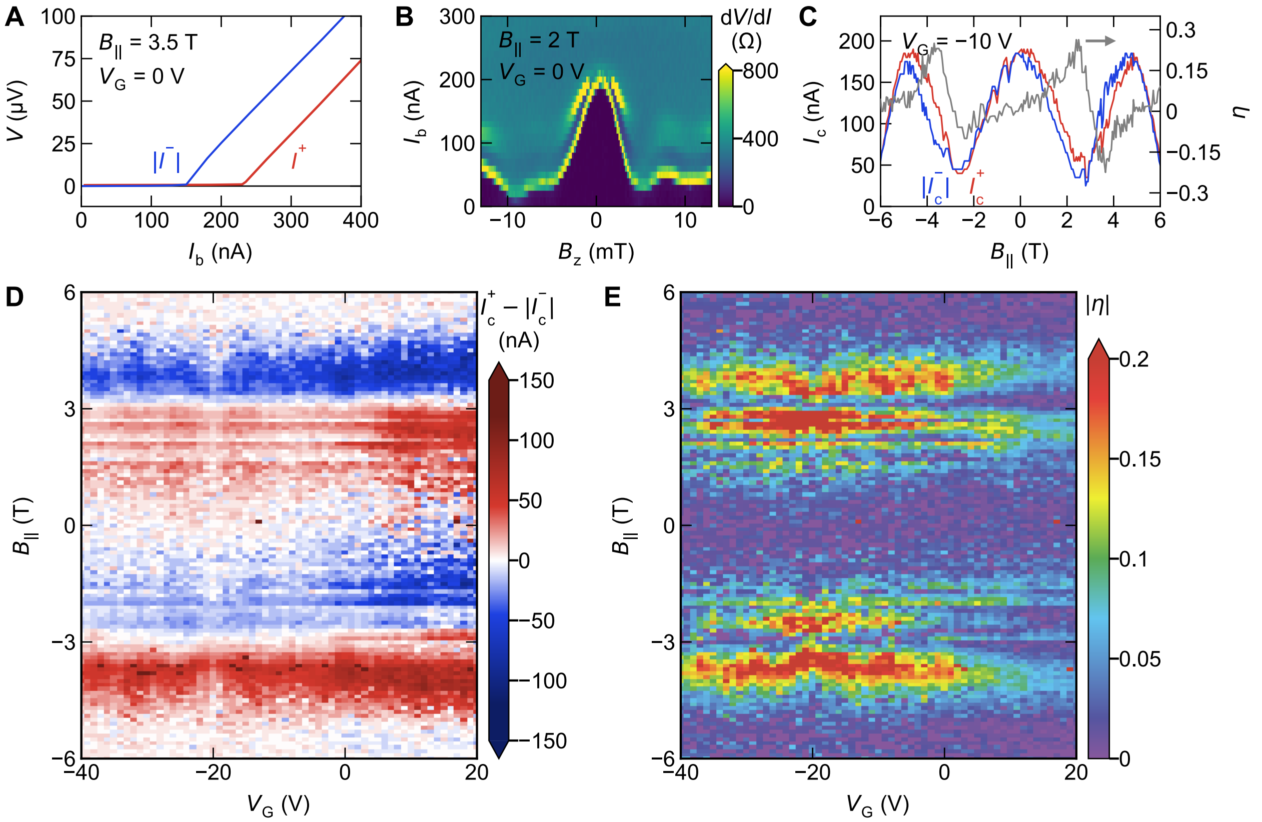

Figure S3: **Reproducibility in device C. A,** I-V characteristics at $B_{||} = 3.5\,\mathrm{T}$ and $V_G = 0\,\mathrm{V}$. The negative bias curve (blue) was flipped horizontally and vertically. The current sweep was always from zero. **B,** Color map of dV/dI as a function of B_z and I_b at $B_{||} = 2\,\mathrm{T}$ and $V_G = 0\,\mathrm{V}$. **C,** I_c for positive bias currents (red) and negative bias currents (blue), as well as the diode efficiency η (grey) as a function of $B_{||}$ at $V_G = -10\,\mathrm{V}$. **D,** Color mapping of $I_c^+ - |I_c^-|$ as a function of V_G and $B_{||}$. **E,** Color mapping of $|\eta|$ as a function of V_G and $B_{||}$. Measurements were at T = 30 mK.

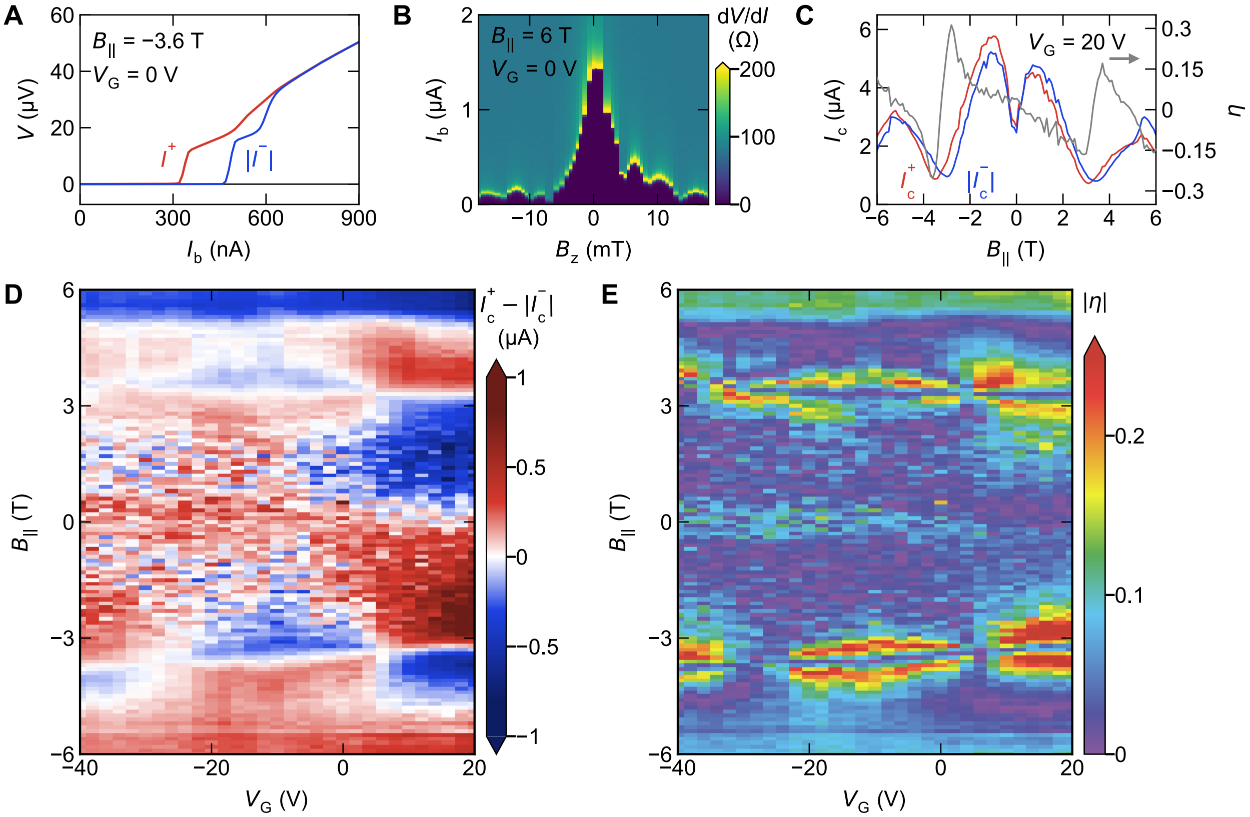

Figure S4: **Reproducibility in device D. A,** I-V characteristics at $B_{\parallel} = -3.6\,\mathrm{T}$ and $V_G = 0\,\mathrm{V}$. The negative bias curve (blue) was flipped horizontally and vertically. The current sweep was always from zero. **B,** Color map of $\mathrm{d}V/\mathrm{d}I$ as a function of B_z and I_b at $B_{\parallel} = 6\,\mathrm{T}$ and $V_G = 0\,\mathrm{V}$. **C,** I_c for positive bias currents (red) and negative bias currents (blue), as well as the diode efficiency η (grey) as a function of $B_{\parallel}$ at $V_G = 20\,\mathrm{V}$. **D,** Color mapping of $I_c^+ - |I_c^-|$ as a function of V_G and $B_{\parallel}$. **E,** Color mapping of $|\eta|$ as a function of V_G and $B_{\parallel}$. Measurements were at $T = 30$ mK.

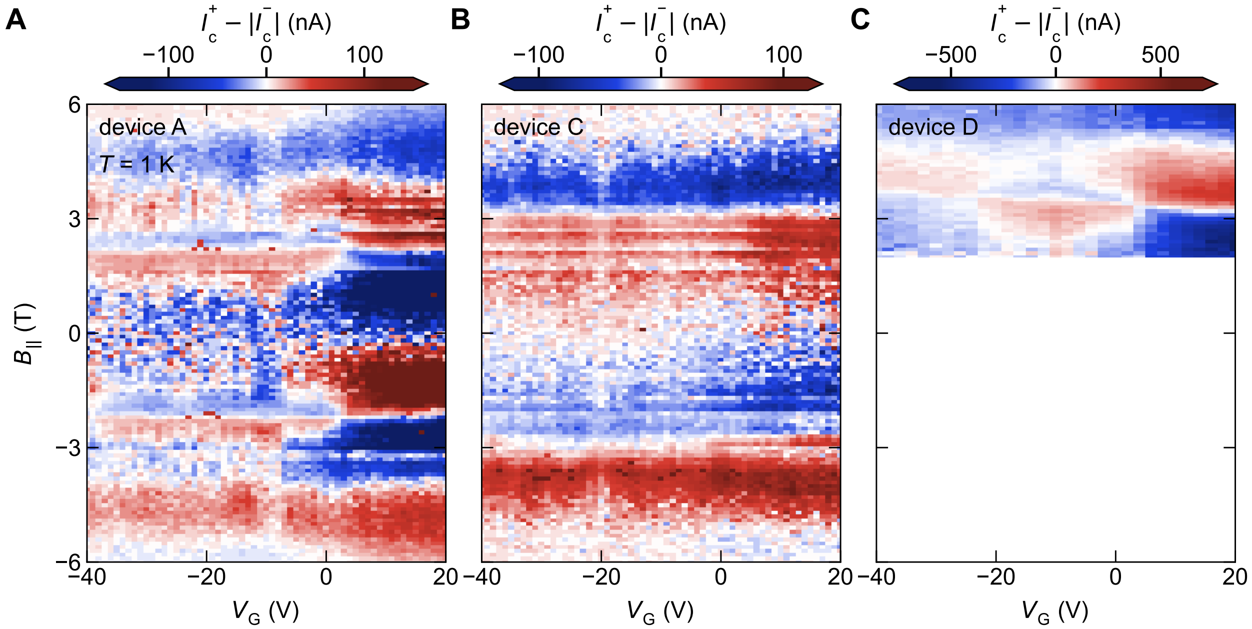

Figure S5: **A, B, C,** Color mapping of $I_c^+ - |I_c^-|$ at $T = 1$ K as a function of V_G and $B_{\|}$ in devices A, C, and D. The measurement of Device D at 1 K was performed only for a limited range of $B_{\|}$, since there was little change from the behavior at 30 mK shown in Fig. S4.

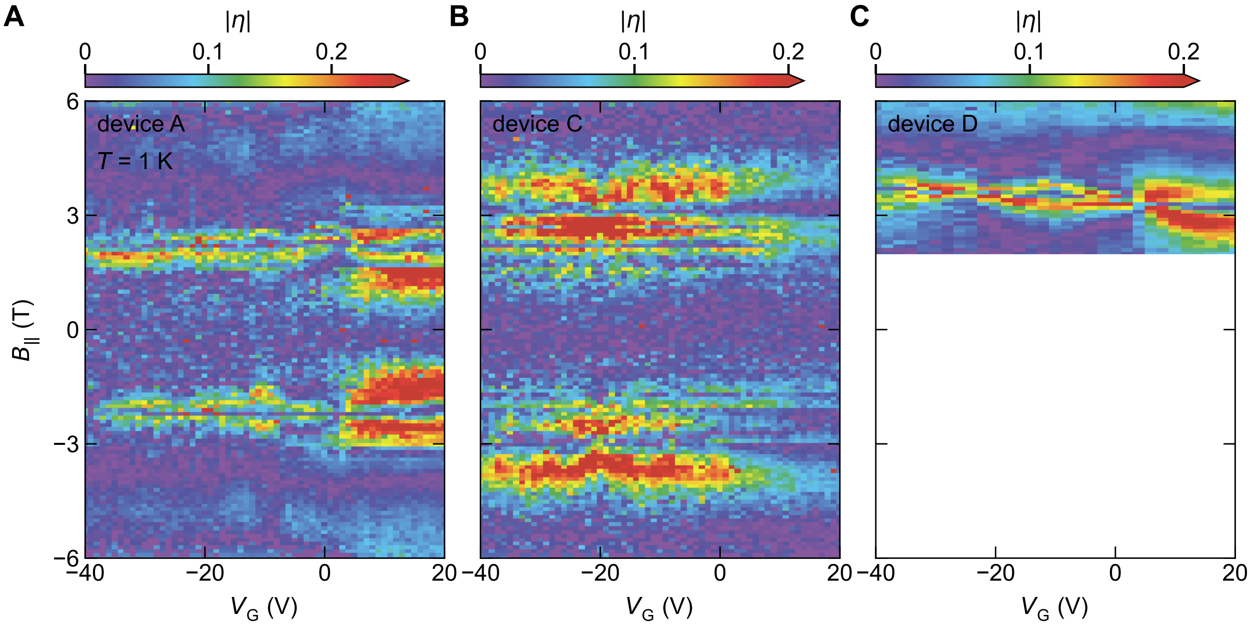

Figure S6: **A, B, C,** Color mapping of $|\eta|$ at $T = 1$ K as a function of V_G and $B_{\parallel}$ in devices A, C, and D.

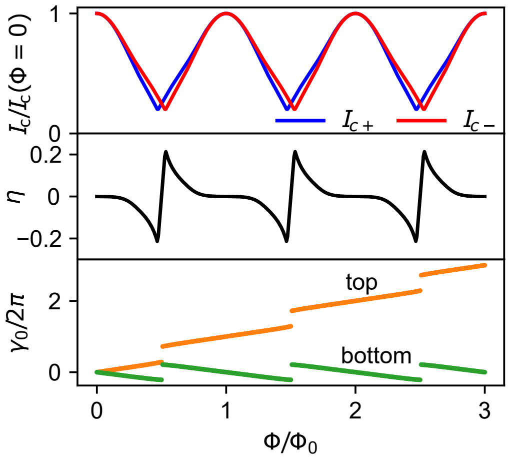

Figure S7: **Phenomenological model**. Critical currents in positive and negative directions $I_{c\pm}$, diode efficiency η, and gauge-invariant equilibrium phase bias $\gamma_{0,t/b} = \theta_0 + \int \mathbf{A}d\mathbf{l} = \theta_0 \pm \pi\Phi/\Phi_0$ at top- and bottom surfaces as a function of magnetic flux. Shown here are the model calculations from Eq. (1) in the main text with skewness $S_t = S_b = -0.2$ and asymmetry $I_{0,t}/I_{0,b} = 0.8$.

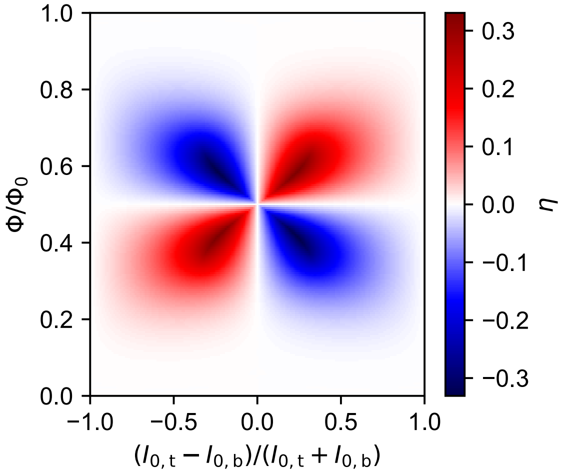

Figure S8: Diode efficiency calculated from Eq. (1) in the main text, as a function of Φ and top-bottom asymmetry $\frac{I_{0,\mathrm{t}}-I_{0,\mathrm{b}}}{I_{0,\mathrm{t}}+I_{0,\mathrm{b}}}$. The skewness parameter setting the weight of the second harmonic is $S_\mathrm{t} = S_\mathrm{b} = -0.2$.

Device	d (nm)	W (nm)	L (µm)
A	15	60	1.2
B	22	80	2.4
C	13	60	1.5
D	12	70	4.9

Table S1: **Nanowire dimensions for devices A–D.**
